# Absence of transmission of NDM and OXA-48 carbapenemase genes in a chronic care unit of a long-term care facility

**DOI:** 10.1177/17571774211012443

**Published:** 2021-06-22

**Authors:** Carl Boodman, Natalie Gibson, Davenna Conrod, Christine Y Turenne, David C Alexander, Tatyana Taubes, Ana Lucha, David A Boyd, Laura F Mataseje, Michael Mulvey, James A Karlowsky, Molly Blake, John M Embil

**Affiliations:** 1 Department of Internal Medicine, University of Manitoba, Winnipeg, Canada; 2 Department of Medical Microbiology and Infectious Diseases, University of Manitoba, Winnipeg, Canada; 3 Infection Prevention and Control Program, Winnipeg Regional Health Authority, Winnipeg, Canada; 4 Shared Health, Winnipeg, Canada; 5 Cadham Provincial Laboratory, Winnipeg, Canada; 6 Deerlodge Centre, Winnipeg, Canada; 7 National Microbiology Laboratory, Public Health Agency of Canada, Winnipeg, Canada

**Keywords:** New Delhi metallo-β-lactamase (NDM), oxacillinase (OXA)-48, carbapenemase, long-term care, chronic care

## Abstract

Infection prevention and control measures are used to contain outbreaks of carbapenemase-producing Enterobacteriaceae. We report the absence of transmission of *Klebsiella pneumoniae* carrying New Delhi metallo-β-lactamase and oxacillinase-48 genes among 19 screened contacts of an index case after 14 months of routine practices in a long-term care facility.

## Background

The emergence of carbapenem resistance among Gram negative bacteria heralds a future of potentially untreatable infections due to extreme antimicrobial resistance (Paterson and Doi, 2007). The hydrolysis of carbapenems by carbapenemase-producing Enterobacteriaceae (CPE) is an emerging mechanism of resistance in hospital-acquired infections. There are limited antimicrobial treatments existing for patients infected with these pathogens (Tacconelli et al, 2018). Infections due to CPE are associated with greater mortality than those caused by carbapenem-susceptible bacteria (Falagas et al, 2014).

Canadian rates of CPE colonisation increased nearly five-fold from 2014 to 2017; however, an increase in the rate of CPE infection has not yet been documented in Canada (Public Health Agency of Canada, 2019). The implementation of institutional control measures is deemed essential to limit the spread of CPE locally, nationally and internationally (French et al, 2017; Public Health Agency of Canada, 2019).

Combined control measures are typically required to prevent and control outbreaks with CPE. These measures include routine practices and additional precautions such as contact precautions, hand hygiene, staff education, patient isolation, enhanced environmental cleaning and disinfection, and active surveillance (French et al, 2017). Considering that silent CPE transmission has occurred in the context of contact isolation, identifying asymptomatic patients colonised with CPE through active surveillance is essential to curtail outbreaks (French et al, 2017). Diligent use of additional precautions and appropriate equipment cleaning and disinfection for patients acting as asymptomatic reservoirs has led to successful cluster extinction (Calfee and Jenkins, 2008). Considering the presumed prolonged colonisation with CPE, most public health guidelines suggest that contact precautions be kept indefinitely. The longevity of colonisation is now being questioned (Tucker et al, 2019).

Here, we report the findings of a programme of active contact surveillance for a CPE positive index case who had been residing in the chronic care unit of a long-term care facility (LTCF) for 14 months following the isolation of CPE during a brief hospital admission. The patient’s status as an individual infected or colonised with a CPE was not known to the chronic care unit. As a result, only routine practices were implemented during this time.

## Methods

This study was performed at the chronic care unit of a LTCF within Winnipeg, Manitoba, Canada. Subjects were patients who were in contact with a CPE colonised individual prior to the initiation of specific infection prevention and control (IPC) measures. Subjects were screened for CPE colonisation via rectal swabs collected on days 0, 7 and 21 after exposure was identified. Swabs were cultured on CHROMagar mSuperCARBA agar (CHROMagar, Paris, France), and Gram negative, carbapenem-resistant organisms were identified to species level using matrix-assisted laser desorption/ionisation-time of flight mass spectrometry (MALDI-TOF MS; Bruker Daltonics, Billerica, MA, USA). Phenotypic confirmation of carbapenemase production by Enterobacteriaceae isolates was performed using the Neo-Rapid CARB kit (Rosco Diagnostica; Taastrup, Denmark), a chromogenic assay based on the hydrolysis of imipenem. The carbapenemase type was initially determined by a polymerase chain reaction (PCR) screen capable of detecting *KPC*, *NDM*, *VIM*, *OXA-48*, *IMP* and *GES* genes. Phenotypic susceptibility testing was performed on all CPE isolates using the Vitek 2 N208 or N391 cards (bioMérieux, St-Laurent, Québec, Canada) and disk diffusion and interpreted according to current Clinical and Laboratory Standards Institute (CLSI) and US Food and Drug Administration (FDA) breakpoints (for tigecycline). Whole genome sequencing of carbapenemase-producing Enterobacteriaceae was performed with the Illumina MiSeq platform (Illumina, San Diego, CA, USA). Sequencing data have been deposited in NCBI as BioProject PRJNA511988 and BioSamples SAMN13659125 (*Klebsiella pneumoniae*) and SAMN13659126 (*Escherichia coli*). Contigs were generated using the assembly and annotation pipeline of the rapid infectious disease analysis platform (IRIDA v19.09), which combines SPAdes-based de novo assembly with Prokka-based annotation. Carbapenemase-producing genes were characterised using ResFinder 3.2 (Center for Genomic Epidemiology, https://cge.cbs.dtu.dk/services/) and CARD (the comprehensive antibiotic resistance database, https://card.mcmaster.ca/home).

Research ethics approval for retrospective chart review was approved as well as institutional approval at the LTCF.

## Results

Our index patient was first found to have a CPE-producing isolate of *K. pneumoniae* from a urine sample obtained in May 2017 during an acute care hospitalisation. As per regional practice, this patient’s CPE status was identified in the electronic patient record (EPR) to ensure that staff are aware of the need for additional infection control measures. This organism demonstrated phenotypic antimicrobial susceptibility only to tigecycline (Table 1). Carbapenemase activity was confirmed phenotypically and by PCR detection of both *NDM* and *OXA-48* genes. During repeat acute hospital admissions in February 2018 and May 2019, two additional urine isolates from this same index case demonstrated the presence of *K. pneumoniae* containing both *NDM* and *OXA-48* carbapenemases. Our patient was admitted in April 2018 from the community to a chronic care unit at a LTCF in Winnipeg where this individual lived in a single room. This facility did not have access to the EPR system, so the patient’s CPE status was unknown to the site. During temporary admission to hospital in August 2019, a blood culture from the index case yielded a CPE isolate of *Klebsiella aerogenes.* All urine and blood samples were obtained during repeated acute care hospitalisations. Between admissions, our patient returned to the chronic care unit of the same LTCF.

**Table 1.** Summary of index and contact isolates including date, specimen type, species identification and antimicrobial susceptibility testing.

	1st isolate from index case	2nd isolate from index case	Isolate from contact
Date of isolation	May 2017	May 2019	June 2019
Specimen	Urine	Urine	Rectal swab
Organism	*K. pneumoniae*	*K. pneumoniae*	*E. coli*
Carbapenemase genotype	NDM, OXA-48	NDM, OXA-48	OXA-181
Ampicillin	R (⩾32 μg/mL)	R (⩾32 μg/mL)	R (⩾32 μg/mL)
Amoxacillin-clavulanic acid	R (⩾32 μg/mL)	R (⩾32 μg/mL)	R (⩾32 μg/mL)
Piperacillin-tazobactam	R (⩾128 μg/mL)	R (⩾128 μg/mL)	R (⩾128 μg/mL)
Cefazolin	R (⩾64 μg/mL)	R (⩾64 μg/mL)	R (⩾64 μg/mL)
Ceftriaxone	R (⩾64 μg/mL)	R (⩾64 μg/mL)	R (16 μg/mL)
Ceftazidime	R (⩾64 μg/mL)	R (⩾64 μg/mL)	R (15 mm)
Meropenem	R (⩾32 μg/mL)	R (⩾32 μg/mL)	I (21 mm)
Ertapenem	R (6 mm)	R (⩾8 μg/mL)	R (17 mm)
Ciprofloxacin	R (⩾4 μg/mL)	R (⩾4 μg/mL)	R (⩾4 μg/mL)
Gentamycin	R (⩾64 μg/mL)	R (⩾64 μg/mL)	S (⩽1 μg/mL)
Tobramycin	R (⩾16 μg/mL)	R (⩾16 μg/mL)	S (⩽1 μg/mL)
Amikacin	R (⩾64 μg/mL)	R (⩾64 μg/mL)	S (⩽2 μg/mL)
Nitrofurantoin	R (⩾512 μg/mL)	R (⩾512 μg/mL)	S (⩽16 μg/mL)
TMP-SMX	R (⩾320 μg/mL)	R (⩾320 μg/mL)	S (⩽20 μg/mL)
Minocycline	I (8 μg/mL)	I (8 μg/mL)	ND
Tigecycline	S (2 μg/mL)	S (2 μg/mL)	ND
Colistin	I (1 μg/mL)	R (4 μg/mL)	ND
Ceftolozane-tazobactam	R (⩾128 μg/mL)	R (⩾128 μg/mL)	ND
Fosfomycin	ND	(16 μg/mL)^a^	ND

Interpretive categories for each antimicrobial agent are reported with either the MIC (in μg/mL) or disk diffusion zone sizes (in mm) and interpreted according to Clinical and Laboratory Standards Institute (CLSI) breakpoints.

ND: not done; R: resistant; S: susceptible; I: intermediate.

a CLSI breakpoints have not been established for this bacteria/antimicrobial agent combination.

Rectal screening for CPE carriage of 19 contacts of the index case was completed three times between 10 June and 1 July 2019. Over 2 years elapsed between the first laboratory documentation of a CPE and notification of the CPE status to the chronic care unit – 14 months following admission to the chronic care unit. During this 14-month period, the patient was on a unit following routine practices (standard precautions) without additional precautions or enhanced environmental disinfection.

Of the 19 screened contacts, one was colonised with a carbapenemase-producing strain of *E. coli* which was of intermediate susceptibility to meropenem (Table 1). *OXA-48* was detected by PCR analysis. This led to another 11 contacts identified and screened for rectal carriage of CPE between 26 June and 17 July 2019. CPE was not recovered. A fourth screening for rectal carriage of CPE was completed on the initial 18 negative index unit contacts in October 2019 and CPE was not identified.

Whole genome sequencing (WGS) was undertaken of the index case’s *K. pneumoniae* urinary isolates (May 2017 and May 2019) and *K. aerogenes* blood isolate (August 2019) as well as the positive CPE contact’s *E. coli* rectal isolate (June 2019). Analyses revealed that the contact’s *E. coli* isolate harbored *OXA-181*, not the *OXA-48* or *NDM* of the index case’s isolates. This established a lack of CPE transmission between the index case and contact. The *OXA-181* enzyme shares 94% nucleotide identify with *OXA-48* and cross-reacts with primers for PCR-based detection of *OXA-48*.

Once the patient’s CPE status was known to the LTFC, specific IPC precautions were immediately initiated. Staff were required to glove and gown prior to entry into the patient environment and to use dedicated equipment. If this was not possible, shared equipment was disinfected after use. Enhanced cleaning and disinfection was implemented on the unit. Education about CPE pathogens was provided to staff, residents and families. Admission screening policies were enhanced so that similar situations do not occur in the future.

## Discussion

Our findings call into question the efficiency of CPE transmission. In our case, routine practices appear to have prevented CPE plasmid and/or clonal spread, a surprising result for a chronic care unit in a LTCF facility where residents share common spaces with other occupants.

Institutional control of CPE typically involves bundled interventions. Thus, it is difficult to tease apart the relative effect of each intervention. It has been noted that routine practices are insufficient in controlling CPE transmission considering previous documented failures of CPE control with the implementation of contact precautions and isolation (Calfee and Jenkins, 2008; French et al, 2017).

The spontaneous decolonisation of CPE colonised patients may provide an explanation for our results (Vink et al, 2019; Zimmerman et al, 2013). Considering that our screening protocol was implemented up to 14 months after initial contact with the CPE positive index case, it remains possible that contacts who were initially CPE positive spontaneously eliminated their CPE by the time of testing (Vink et al, 2019). While a mean time to CPE negativity of approximately one year has been described among CPE colonised patients after hospital discharge, further studies are necessary to understand the process of spontaneous decolonisation during continuous CPE exposure (Bar-Yoseph et al, 2016; Zimmerman et al, 2013).

Repeat hospitalisation is known to prolong CPE colonization (Zimmerman et al, 2013). Considering that hospitalisation often involves antibiotic administration, CPE’s transmissibility and duration of detection may be affected by the antimicrobial selection pressure and dysbiosis caused by broad-spectrum antibiotics (Zimmerman et al, 2013). Transfer from a chronic care unit to an acute care facility often involves antibiotic initiation or broadening. Thus, CPE transmissibility may be contextual, dependent on institutional patterns of antibiotic administration.

## Conclusion

While bundled interventions are typically required to control the spread of CPE, we report the absence of transmission of *NDM* and *OXA-48* genes from a CPE index case on a chronic care unit of a LTCF after 14 months of routine practices with no additional precautions. Our findings suggest that CPE transmissibility may differ between institutional settings. Further studies involving genotypic CPE analysis, institutional antimicrobial prevalence and prolonged surveillance sampling of contacts are required to inform whether infection control measures should be tailored to the specific CPE type and institutional context.

## Supplemental Material

sj-pdf-1-bji-10.1177_17571774211012443 – Supplemental material for Absence of transmission of NDM and OXA-48 carbapenemase genes in a chronic care unit of a long-term care facility Click here for additional data file. Supplemental material, sj-pdf-1-bji-10.1177_17571774211012443 for Absence of transmission of NDM and OXA-48 carbapenemase genes in a chronic care unit of a long-term care facility by Carl Boodman, Natalie Gibson, Davenna Conrod, Christine Y Turenne, David C Alexander, Tatyana Taubes, Ana Lucha, David A Boyd, Laura F Mataseje, Michael Mulvey, James A Karlowsky, Molly Blake and John M Embil in Journal of Infection Prevention
