# Supplementary material for: Absence of transmission of NDM and OXA-48 carbapenemase genes in a chronic care unit of a long-term care facility
Source: J Infect Prev. 2021 Jun 22;23(1):15–9. doi: 10.1177/17571774211012443 (PMC8811232; doi:10.1177/17571774211012443)
Supplement: sj-pdf-1-bji-10.1177_17571774211012443 – Supplemental material for Absence of transmission of NDM and OXA-48 carbapenemase genes in a chronic care unit of a long-term care facility [file sj-pdf-1-bji-10.1177_17571774211012443.pdf]

Supplementary References for PCR and WGS methods:

PCR assay:

Mataseje LF, Bryce E, Roscoe D, et al. Carbapenem-resistant Gram-negative bacilli in Canada 2009–10: results from the Canadian Nosocomial Infection Surveillance Program (CNISP). *J Antimicrob Chemother*. 2012;67:1359-1367.

Integrated Rapid Infectious Disease Analysis (IRIDA) Platform:

Matthews TC, Bristow FR, Griffiths EJ, et al. The Integrated Rapid Infectious Disease Analysis (IRIDA) Platform. *bioRxiv*. January 2018:381830.

ResFinder 3.2 (Centre for Genomic Epidemiology):

Zankari E, Hasman H, Cosentino S, et al. Identification of acquired antimicrobial resistance genes. *J Antimicrob Chemother*. 2012;67:2640-2644.

The Comprehensive Antibiotic Resistance Database (CARD):

McArthur AG, Waglechner N, Nizam F, et al. The Comprehensive Antibiotic Resistance Database. *Antimicrob Agents Chemother*. 2013;57:3348 LP - 3357.
